# Dissociation Constant of Anisic (*p*-Methoxybenzoic) Acid in the System Ethanol-Water at 25 °C

**DOI:** 10.6028/jres.064A.035

**Published:** 1960-08-01

**Authors:** Elizabeth E. Sager, Vincent E. Bower

## Abstract

The dissociation constant of anisic (*p*-methoxybenzoic) acid in the system ethanol-water was measured at 25° C by a combination of spectrophotometric and electrometric methods. Percentage by weight of ethanol in the solvent system varied from zero to 83.43. The value of *K* varied correspondingly from 3.22×10^−5^ (*pK*=4.492) to 3.34×10^−8^ (*pK* = 7.476).

## 1. Introduction

A new phase in the technique of measuring dissociation constants of weak acids dates from 1932 when Harned and Ehlers [1] used^1^ measurements of the cell Pt; H_2_(g, 1 atm), CH_3_ COOH (*m*_1_), CH_3_ COONa (*m*_2_), NaCl (*m*_3_), AgCl (*s*); Ag to determine the dissociation constants of acetic acid from 0° to 60° C. In the same year MacInnes and Shedlovsky [2] used conductance measurements to determine the dissociation constant of this acid at 25° C. Since then an immense body of factual information has become available on the dissociation constants of weak acids in aqueous solution. In many instances the variation of the dissociation constant with temperature has been studied. The field of nonaqueous solutions and of mixed solvents with water as one component has, however, been comparatively neglected. The dissociation constant of acetic acid has been measured in 20, 45, 70, and 82 percent dioxane [3 to 6]; in 10 percent and 20 percent methanol [7]; in 50 percent glycerol [8]; and, more recently, over the 0 to 100 percent range of water-methanol system [9]. Again, the dissociation constants of the ammonium and the methylammonium ions have been measured in 60 percent methanol [10]. Other examples could be noted, but they are few compared with the number of determinations that have been made in aqueous solution.

As a contribution to this field, measurements on anisic acid (*p*-methoxybenzoic acid) in the ethanol-water system are now reported. This acid was selected because it is available in a state of high purity, and its dissociation constant in water (*p*K*_a_*=4.471 at 25° C) has already been measured [11]. Moreover, there are data in the literature for a buffer system in ethanol-water mixtures in this dissociation range [12].

## 2. Experimental Procedure

### 2.1. Materials

Anisic acid, NBS Standard Sample No. 142, was used without further purification. The white, powdered material is sparingly soluble in water, and an original stock solution, about 2×10^−2^ *M*, was made by dissolving a weighed amount in 95 percent ethanol and warming the resulting solution in a water-bath. A second stock solution was prepared for each series by diluting 5 ml of the original solution to 100 ml with water.

Hydrochloric acid of reagent grade was used to control the hydrogen-ion concentration of the solutions employed to determine the limiting spectrophotometric curves of the anisic acid. Standardized sodium hydroxide, free from carbonate, was used to transform the acid completely to the salt. Purified sodium acetate (hydrate) was employed for the acetate buffers. A 1-*M* stock solution was prepared from which dilutions were made by volumetric procedures. Glacial acetic acid was used to prepare a 1-*M* stock solution, of which varying small amounts ½ to 4 ml, were used to change the buffer ratio, while keeping the acetate constant. Thus the ratio of the anisic acid to its salt could be measured spectrophotometrically.

Potassium chloride, recrystallized three times from water and dried at 110° C, was used to make all solutions 5×10^−3^ *M* with respect to chloride for the electromotive force measurements.

The ethanol was tested for impurities and met ACS specifications. Conductivity water was used throughout for all preparations.

### 2.2. Equipment

A Beckman DU spectrophotometer equipped with a water-jacketed cell compartment was used for the spectrophotometric measurements. The temperature was controlled at 25° C within ±0.05° C. Cylindrical 1-cm absorption cells with removable quartz endplates were used throughout.

Electromotive force measurements of the following cell without liquid junction were made for each ethanol-water mixture.

$${\text{Pt; H}}_{2}(1\;\text{atm}),\;\text{HA}\;(m_{1}),\;\text{NaA}\;(m_{2}),\;\text{KCl}\;(0.005\;m),\;\text{AgCl}(s);\;\text{Ag}$$ (A)

The cell was a simplified version, much reduced in size, of that described by Bates [13, 14]. The volume of the cell is only 10 ml and the solution is therefore quickly saturated with hydrogen. Data required for the correction of the electromotive force of the cells to 1 atm partial pressure of hydrogen were calculated from values of the vapor pressures of alcohol-water system given in International Critical Tables [15].

## 3. Calculation of Dissociation Constants From Spectral Absorbance Data and Electromotive Force Measurements

### 3.1. Absorbance Measurements

Anisic acid shows well-defined absorbance in the ultraviolet. When the acid is transformed to the salt the absorbance shifts to lower wavelengths. The shift, however, is a small one and consequently absorbance values must be read from curves of very steep slope for making the calculations. Also, when alcohol is introduced into the water system, there is a slight hypsochromic effect, both bands for the acid and for the salt shifting toward lower wavelengths. Therefore it was necessary to determine carefully the so-called “limiting” curves which represent the transformation to all acid or to all salt in ethanol-water mixtures. For each series a slightly different isosbestic point exists; thus, the complete spectrum was examined for behavior and stability of each system by extensive measurements throughout the ultraviolet.

The reaction may be simply represented as the acid dissociating to the ions, namely,

$$\text{HA}⇌[H^{+}]+[A^{-}].$$

According to the law of absorption, at any given wavelength,

Molar absorbance=A/(bM), (1)

in which *A* is the absorbance of the sample (−log transmittancy), *b* is the depth of solution in centimeters through which the radiant energy passes, and *M* is the molar concentration of the absorbing compound. Molar absorbance is expressed in liters mole^−1^ cm^−1^.

**Figure 1:**
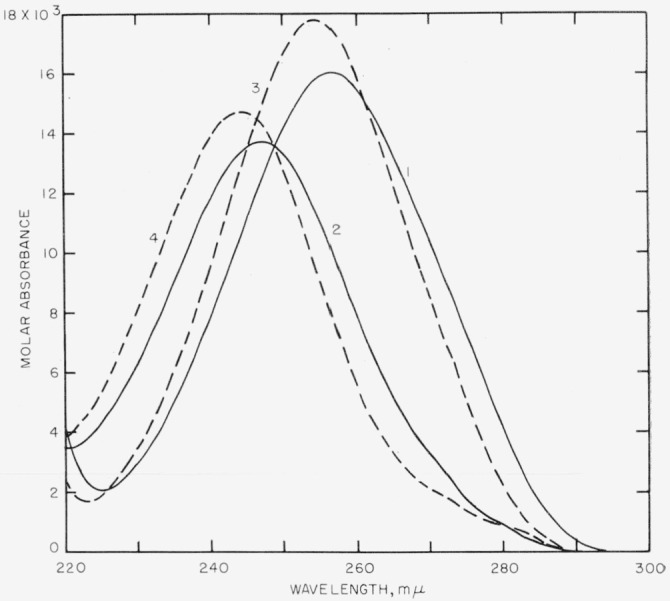
The limiting curves of anisic acid and its salt in water (curves 1, 2) and in 83.43 percent by weight ethanol (curves 3, 4).

The values of molar absorbance of anisic acid and its sodium salt are shown in figure 1, the solid lines 1 and 2 representing the compound in water, and the broken lines 3 and 4 representing the material in 83.43 percent by weight of ethanol.

**Figure 2:**
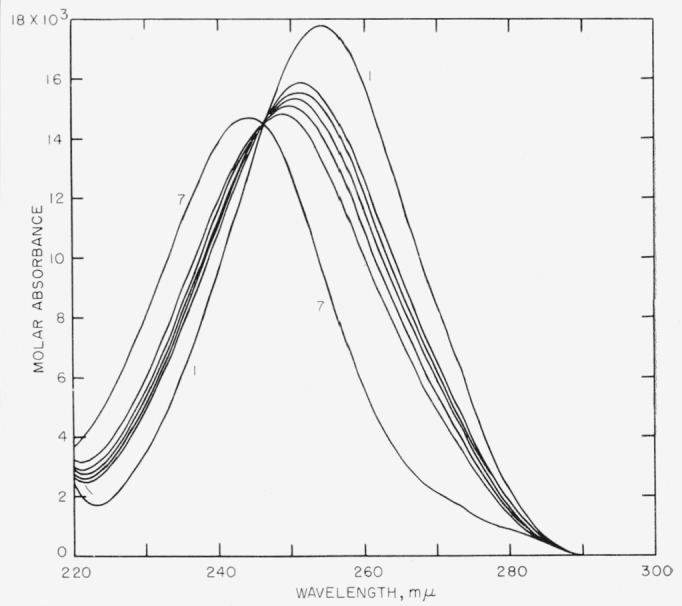
Spectrophotometric curves representing the dissociation of anisic acid in 83.43 percent ethanol by weight. Curve 1, Anisic acid; curve 7, the sodium salt.

In water the acid shows maximum molar absorbance of about 16,000 liters mole^−1^ cm^−1^ at a wavelength of 257 m*μ* and the salt shows a maximum of about 13,000 liters mole^−1^ cm^−1^ at 247 m*μ.* The two species have the same absorption at 249 m*μ*, the isosbestic point. In 83.43 percent ethanol by weight, the maximum molar absorbance of the acid increases to 17,800 liters mole^−1^ cm^−1^, at 254.6 *mμ*, while the maximum of the salt increases to 14,700 liters mole^−1^ cm^−1^, at 244.2 m*μ*, with the isosbestic point at 246.4 m*μ.* Figure 2 shows the series of curves for different degrees of dissociation of anisic acid in 83.43 percent ethanol by weight. It is typical of the other series studied, but in the illustrated series the slopes of the curves are steeper and therefore involve greater experimental errors than in the other series. All series exhibited well-defined isosbestic points.

### 3.2. Calculation of Ratio of Salt to Acid

In any given series, the amounts of undissociated acid and of the salt are proportional to their respective absorbance values. If *α* represents the amount of salt, then at any step in the dissociation,

$$\alpha =\frac{A_{\text{HA}}-A\alpha }{A_{\text{HA}}-A_{A-}},$$ (2)

in which *A* with appropriate subscripts represents the absorbance of the acid, the absorbance of the partially dissociated acid, and the absorbance of the salt. The ratio *α*/(1−*α*) may be calculated directly, but when the absorbance values are taken from such steep curves where experimental errors are large, it is wise to examine the *α* values at several wavelengths so that any trend in the values or influence of the buffering system may be determined. For this reason an average *α* at 16 wavelengths was used and the ratio *α*/(1−*α*) calculated afterwards.

### 3.3. Calculation of *p*K

After the spectrophotometric observations were completed, each solution and its companion buffer solution were measured by electromotive force methods.

The *p*K of anisic acid on the thermodynamic scale is given by

$$pK=-\log[H^{+}]-\log\frac{\alpha }{1-\alpha }-\log\frac{\gamma_{A-}}{\gamma_{\text{HA}}},$$ (3)

where *γ* represents the activity coefficient of the ionic or molecular species denoted by the subscripts. For a cell of type *A*

$$-\log[H^{+}]\gamma_{H}\gamma_{\text{Cl}}=(E-E{}^{\circ})/(2.303RT/F)+\log m_{\text{Cl}}+\log\gamma_{\text{Cl}},$$ (4)

where *R*, *T*, and *F* are, respectively, the gas constant, the absolute temperature, and the faraday, and where *E°* is the standard potential of the silver-silver chloride electrode in the particular alcohol-water system [16].

By combination,

$$pK=pwH-\log\frac{\alpha }{1-\alpha }-\log\frac{\gamma_{A-}}{\gamma_{\text{Cl}}-\gamma_{\text{HA}}},$$ (5)

which is essentially the method of calculation employed by Bates and Schwarzenbach [17]. Inasmuch as the last term of eq (5) is approximately zero at low and moderate ionic strengths eq (5) simplifies to

$$pK_{a}\approx pwH-\log\frac{\alpha }{1-\alpha }.$$ (6)

The calculations of the dissociation constant of anisic acid in water and in nine ethanol-water mixtures are shown in table 1. In column 1 the weight percent of ethanol is given and in column 2 the average *α* for each solution as determined by the spectrophotometric measurements. Values of log *α*/(l−*α*) are in column 3 and values of *pw*H, from electromotive force measurements, are listed in column 4. From columns 3 and 4 the *p*K of anisic acid is derived as shown in column 5 with the average for each series in column 6. As the dielectric constant of each mixture decreases, the value of *p*K increases as the ethanol content increases.

It was not our intention to determine the dissociation constants of acetic acid in the ethanol-water system since it has already been reported [1]. The ratio of acetate to acetic acid was known from the volumetric preparation of the buffer systems, but because the amounts of acetate and acetic acid added by pipet are small, the experimental error is expected to be large. Electromotive force measurements were made on the buffering solutions with and without anisic acid. At the concentrations used, the anisic acid had no observable effect upon *pw*H. From the *emf* measurements, then, the *p*K of acetic acid could be calculated according to the relation:

$$pK=pwH-\log\frac{\text{salt}}{\text{acid}}$$ (7)

The buffer ratios are shown in column 7 with the corresponding values of −log (salt/acid) in column 8. The *p*K values of acetic acid in the same series of ethanol-water system are given in column 9 and averaged in column 10. Our data show slightly higher values than those found by Grunwald and Berkowitz, but it should be noted that the values for both anisic acid and for acetic acid in water are in reasonable agreement with published values.

To test the validity of eq 7, by which Bates and Schwarzenbach found that *p*K*_a_* was independent of ionic strength in the case of a buffering system of this charge type, several experiments were made using equimolar solutions of acetate-acetic acid buffer at 0.015, 0.025, and 0.055 ionic strengths in several ethanol-water mixtures. The resulting absorbance curves were the same within the experimental error, and the *pw*H values also agreed within the experimental error. The fact that *p*K*_a_* remains constant, as the ionic strength is varied, suggests the soundness of the assumption that log *γ_Cl_*_−_ and log *γ_A_*_−_ are representable by the same function. It also reveals the use of the acidity function *pw*H in combination with spectrophotometric measurements to be the valid and promising method that Bates and Schwarzenbach contend it to be.

## Figures and Tables

**Table 1:** Dissociation of anisic acid in ethanol-water systems with acetic acid-sodium acetate buffers

Wt. % ethanol	Averaged *α* in %	log *α*/(1−*α*)	*pw*H	*p*K of Anisic acid, (*pw*H-log *α*/(1−*α*))	Average *p*K	Buffer ratio, $\frac{\text{salt}}{\text{acid}}$	Log $\frac{\text{salt}}{\text{acid}}$	*p*K of acetic acid (*pw*H-log salt/acid)	Average *p*K
									
0.00	48.2	−0.032	4.456	4.488		0.02/.04	−0.301	4.757	
	54.9	.085	4.590	4.505		.02/.03	−.176	4.766	
	65.1	.270	4.760	4.490		.01/.01	.000	4.760	
	65.3	.274	4.762	4.488		.02/.02	.000	4.762	
	65.0	.268	4.762	4.494		.05/.05	.000	4.762	
	79.0	.575	5.063	4.488	4.492	.02/.01	.301	4.762	4.762
7.64	53.0	.051	4.706	4.655		.02/.03	−.176	4.882	
	57.1	.124	4.799	4.675		.02/.025	−.097	4.896	
	62.3	.218	4.873	4.655		.02/.02	.000	4.873	
	76.7	.517	5.152	4.635	4.655	.02/.01	.301	4.851	4.874
15.43	42.1	−.139	4.712	4.851		.02/.04	−.301	5.013	
	49.1	−.016	4.836	4.852		.02/.03	−.176	5.012	
	59.2	.161	5.013	4.852		.02/.02	.000	5.013	
	74.4	.463	5.304	4.841	4.849	.02/.01	.301	5.003	5.010
23.28	40.7	−.164	4.991	5.155		.02/.03	−.176	5.167	
	45.5	−.079	5.065	5.144		.02/.025	−.097	5.162	
	51.9	.033	5.165	5.132		.02/.02	.000	5.165	
	58.3	.145	5.283	5.138		.02/.015	.124	5.159	
	67.1	.309	5.466	5.157	5.145	.02/.01	.301	5.165	5.163
31.58	30.2	−.365	5.048	5.413		.02/.04	−.301	5.349	
	36.0	−.251	5.169	5.420		.02/.03	−.176	5.345	
	46.2	−.067	5.352	5.419		.02/.02	.000	5.352	
	62.3	.218	5.652	5.434		.02/.01	.301	5.351	
	76.7	.517	5.925	5.408	5.419	.02/.005	.602	5.323	5.344
40.13	31.1	−.346	5.364	5.710		.02/.03	−.176	5.540	
	41.1	−.157	5.505	5.662		.02/.02	.000	5.505	
	48.1	−.034	5.673	5.707		.02/.015	.124	5.549	
	57.9	.138	5.850	5.712		.02/.01	.301	5.549	
	72.7	.425	6.117	5.692	5.697	.02/.005	.602	5.515	5.531
49.18	29.6	−.377	5.602	5.979		.02/.03	−.176	5.778	
	38.6	−.203	5.773	5.976		.02/.02	000	5.773	
	45.3	−.082	5.895	5.977		.02/.15	.124	5.771	
	55.8	.101	6.069	5.968		.02/.01	.301	5.768	
	71.0	.388	6.371	5.983	5.976	.02/.005	.602	5.769	5.779
58.76	29.5	−.379	5.866	6.245		.02/.03	−.176	6.042	
	38.6	−.202	5.993	6.195		.02/.02	.000	5.993	
	45.3	−.082	6.157	6.239		.02/.015	.124	6.033	
	55.1	.088	6.338	6.250		.02/.01	.301	6.037	
	70.6	.380	6.615	6.235	6.235	.02/.005	.602	6.013	6.020
68.99	29.3	−.383	6.188	6.571		.02/.03	−.176	6.364	
	38.1	−.212	6.376	6.588		.02/.02	.000	6.376	
	44.2	−.102	6.488	6.590		.02/.015	.124	6.364	
	55.0	.087	6.681	6.584		.02/.01	.301	6.380	
	70.1	.369	6.919	6.550	6.577	.02/.005	.602	6.314	6.359
83.43	30.7	−.354	7.144	7.498		.02/.03	−.176	7.320	
	35.2	−.266	7.285	7.551		.02/.025	−.097	7.382	
	40.5	−.168	7.243	7.411		.02/.02	.000	7.243	
	47.0	−.053	7.410	7.463		.02/.015	.124	7.286	
	55.7	.099	7.556	7.457	7.476	.02/.01	.301	7.255	7.297

## Footnotes

1 Figures in brackets indicate the literature references at the end of this paper.
